# Physical activity habits and their relationship with sociodemographic factors in Chilean adolescents

**DOI:** 10.3389/fpsyg.2022.915314

**Published:** 2022-08-17

**Authors:** Sergio Fuentealba-Urra, Andrés Rubio, Carol Flores-Rivera, Mónica González-Carrasco, Juan Carlos Oyanedel, Humberto Castillo-Quezada, Cristian Céspedes-Carreño, Jaime Pacheco-Carrillo

**Affiliations:** ^1^Facultad de Educación y Ciencias Sociales, Universidad Andres Bello, Santiago, Chile; ^2^Facultad de Economía y Negocios, Universidad Andres Bello, Santiago, Chile; ^3^Faculty of Psychology, Diego Portales University, Santiago, Chile; ^4^Research Institute on Quality of Life, University of Girona, Girona, Spain; ^5^Departamento de Ciencias de la Educación, Universidad del Bio-Bio, Concepción, Chile

**Keywords:** physical activity programs, public policy, lifestyles, physical education, social vulnerability, schoolchildren, public policies

## Abstract

Physical activity plays an important role in the well-being and development of adolescents. Physical activity habits expressed in terms of frequency and duration are consistently associated with sociodemographic factors such as age, gender, and socioeconomic status. However, there is less evidence of the relationship between the type and context of physical activity in adolescents. The aim of this article is to analyze physical activity habits and their relationship with sociodemographic factors in Chilean adolescents. The cross-sectional study consisted of 7,263 adolescents aged between 10 and 20 years old, students from both public and private schools in all regions of Chile. Physical activity habits were examined by means of a self-report questionnaire. The age groups were classified according to the three stages of adolescence (early: 10 to 13, middle: 14 to 16, and late: 17 to 20 years old). Socioeconomic level was established based on the school vulnerability index (SVI) of the school attended by each adolescent. In the study it was obvious to the level of physical activity for the adolescents was below the international recommendations. A statistically significant association can also be found between the sociodemographic factors studied and the physical activity habits reported by the young people. The multivariate regression analysis established that the risk of not achieving the physical activity recommendations was 2.8 times higher in females than in males, 2.4 times higher in the older age groups (14–16 and 17–20 years old) compared to the 10–13-year age range and 1.1 times in the medium and high vulnerability groups than in the low socioeconomic vulnerability group. These findings highlight the importance of considering all these factors holistically whenever designing programs or public policies that promote the development of healthy physical activity habits in adolescents.

## Introduction

Adolescence is recognized as a stage in which individuals undergo important physical, psychological and emotional changes (Jiménez Boraita et al., 2020). At this stage in particular, the person moves toward a greater degree of autonomy in relation to their lifestyle, particularly with habits related to a healthy lifestyle (López-Gil et al., 2020). An important part of these habits is related to the regular participation in physical activity, which is critical in the adolescence period since it corresponds to a crucial stage where healthy active habits are acquired and to be consolidated in adulthood (Murphy et al., 2016). Gardner et al. (2011, p. 175) define habits as “behavioral patterns learned through context dependent repetition: repeated performance in unvarying settings reinforces context-behavior associations such that, subsequently, encountering the context is sufficient to automatically cue the habitual response.”

Physical activity corresponds to a behavioral patterns that includes voluntary body movements (Malina and Little, 2008) generated by skeletal muscles that generate significant energy expenditure (Bouchard et al., 1994). In adolescents, physical activity habits are manifested through games, movement, sports, outdoor activities, exercise, and physical education classes (Costigan et al., 2016, 2019). These physical elements are integral to an active lifestyle and correspond to a mediating factor in the relationship between life satisfaction and quality of life of young people (Villafaina et al., 2021). Daily physical activity plays a key role in the improvement and/or maintenance of physical fitness, the prevention of immediate (Vasconcellos et al., 2014) and future physical health problems (Tanné, 2021) and contributes to adequate mental health (Piercy et al., 2018). The World Health Organization (WHO) has developed a series of guidelines related to the practice of physical activity, which suggest that children and adolescents should perform at least 60 min of physical activity daily (World Health Organization [WHO], 2020). Despite the above, the regular practice of PA at this stage is rather insufficient (World Health Organization [WHO], 2014). Globally, approximately 80% of adolescents do not reach the daily physical activity recommendations (Guthold et al., 2020). In Chile, for example, the figure rises to 85% (World Health Organization [WHO], 2017). Similarly, adolescents generally spend two or more hours of their free time in sedentary activities such as watching digital screens (Van Sluijs et al., 2021). The sedentary lifestyle observed in the adolescent population worldwide, has been established as one of the most pressing health challenges facing the world (World Health Organization [WHO], 2017).

In adolescents, physiological, psychological, social and ecological factors may determinate physical activity patterns (Shaw and Shaw, 2014). Sociodemographic factors such as age, socioeconomic status along with gender, are listed as the main factors associated with differences in physical activity patterns (Drenowatz et al., 2010; Luo and Zhong, 2022). Recent studies have reported a significantly lower level of physical activity in adolescent women inside (Rodríguez-Rodríguez et al., 2021) and outside of school (Bennàsser and Vidal-Conti, 2021). Likewise, it has been found that the participation in high-intensity physical activities is lower among women (Langlois et al., 2017). In longitudinal studies, the male gender has been characterized, together with a high socioeconomic level, as non-modifiable factors associated with trajectories that favor healthy physical activity habits (Farooq et al., 2021). Despite the fact that they represent the least studied age group in the literature, it has been observed that in middle and late adolescences there is a considerable decrease in the practice of physical activity in terms of duration and frequency (Best et al., 2017; Van Sluijs et al., 2021). Reviews that have included longitudinal studies from adolescence to adulthood report that it is during adolescence that the most significant decreases in physical activity levels are experienced (Patton et al., 2016; Corder et al., 2019; Hayes et al., 2019).

Additionally, in countries where there are marked socioeconomic disparities amongst the adolescent population, a low socioeconomic level is associated with lower levels of physical activity (Rittenhouse et al., 2011) and the risk of unhealthy physical activity behaviors even interacting with other health risk factors such as overweight and obesity (Jiménez Boraita et al., 2021). Ricardo et al. (2022) who analyzed the physical activity habits of adolescents from Global South Countries, including Chile, reported that even in countries with higher human development indices and higher gender inequality indices, there were significant differences in the levels of physical activity, being consistently lower among women. Precisely, the Global Human Development Report, presented by the United Nations Development Program, indicates that Chile reaches the first place in Latin America and 43rd position among 189 countries worldwide in terms of Human Development Index, however, when this index is adjusted for inequalities, falls to 53rd positions. The report therefore indicates that Chile is a country with marked inequalities in gender and economic prosperity (United Nations Development Programme, 2020).

Considering the said evidence, the aim of this article is to analyze physical activity habits and their relationship with sociodemographic factors in Chilean adolescents. These could also be useful for the following purposes: First, to expand knowledge regarding the frequency and duration of physical activity habits, it can also be used in establishing more precise guidelines for the design of programs and/or public policies to promote the regular practice of physical activity in adolescents, especially amongst the most vulnerable groups (Jiménez Boraita et al., 2021); it could as well be of particular relevance, to analyze physical activity habits considering sociodemographic factors in Chile, a developing country with significant levels of inequality (Mieres Brevis, 2020). We hypothesize that gender, age and socioeconomic level are determining factors in the practice of physical activity and that they constitute, together, predictors of physical activity habits in Chilean adolescents.

## Materials and methods

### Participants and procedures

The sample of this non-experimental cross-sectional study consisted of 7,263 student participants (males = 3,275 [45.1%]; age *M* = 13.88. SD = 2.078) from 5th year of primary school to 4th year of secondary school (school category: public, subsidized and private) across Chile. The sampling was probabilistic, two-stage and stratified (school category and School Vulnerability Index: High, Medium or Low). The first probability sampling unit was the school and then each grade (A, B, C, etc.) within the educational level. The official list of schools 2017, from the Chilean Ministry of Education, was used as the sampling pool. For the year 2017, the number of educational establishments reported by the ministry was 11,749. In that same year, the number of students from fifth to twelfth grade who belonged to the Chilean school system, which corresponds to the population of this study, was 1,863,469. When the educational establishments were contacted, the rate of acceptance to participate in the study was 12.4%. The procedure considered, in the first instance, contacting the school randomly selected to be part of the study. Subsequently, those establishments that decided to participate in the study were evaluated through self-report questionnaires during the 2017 school year, as part of a broader instrument that included other scales. The measurements of the Physical Activity Habits were carried out during class hours, in an optimal classroom environment and under standardized conditions. The instruments of the study were carried out by a group of previously trained individuals in the presence of a teacher from the school.

To establish Physical Activity Habits (PAH), the Eating and Physical Activity Habits Questionnaire for schoolchildren developed and validated by Guerrero et al. (2014) was used (Cronbach’s Alpha, for the entire instrument was 0.81 and 0.76 for the dimension physical activity habits). The said instrument includes two dimensions with a total of 27 items of which 18 are aimed at knowing the eating and nutrition behaviors and 9 are aimed at assessing the physical activity habits of the participants. For this study, only five items specifically assessing physical activity patterns were considered, leaving out those associated with physical inactivity. Questions such as: “Please indicate the frequency with which you perform the following activities: I do physical activities and/or sports with my family,” “I walk at least 15 min per day” (PA derived from commuting) were included. The meaning of physical activity and other terms was clarified previously in each group. Responses range from “daily” (5 points) to “Never or < once a month” (1 point). In order to analyze the structural validity of the construct made up of these 5 items, a Confirmatory Factor Analysis was performed (CFA). The CFA showed an acceptable fit for this model [χ^2^(5, *N* = 7,266) = 242.42, *p* < 0.05; CFI = 0.96, RMSEA = 0.08, SRMR = 0.03] (Hooper et al., 2008). Additionally, this portion of the scale’s Cronbach’s Alpha reliability score was.71, which is regarded as satisfactory (George and Mallery, 2020).

“School Vulnerability Index” (SVI) was used to establish the socioeconomic status (SES) of the participants and corresponds to the percentage of the total number of students (0 to 100%) of a school, which presents conditions of social vulnerability. This index is established based on certain characteristics such as family income, access to and type of housing, number and members of the family group and other social factors such as the educational level of the parents, information contained in Chilean institutions such as the National Civil Registry, the National Health Fund (FONASA) and the Social Protection Card. The SVI can be categorized in three levels [low, medium, or high] according to the percentage of students that each school has qualifying as vulnerable according to the criteria of the National Board of School Aid and Scholarships (JUNAEB). This index corresponds to an indirect measure of SES and has been systematically considered in studies with schoolchildren in the country and is considered reliable to establish such SES (Oyanedel et al., 2015). Our study categorized students who belonged to school with a high SVI as more vulnerable. To establish gender, two options were given to respond (male/female). Finally, to determine the age group, participants were asked their age at the time of answering the questionnaire. After that, the subjects were grouped into three groups, according to the three stages of adolescence described by United Nations International Children’s Fund (UNICEF); early adolescence from 10 to 13 years old, middle adolescence from 14 to 16 years old, and late adolescence from 17 to 21 years old (United Nations International Children’s Fund, 2021).

### Data analysis

The data were described in terms of frequencies for categorical variables and as mean and standard deviation for continuous variables. The relationship of sociodemographic factors with the physical activity habits examined was analyzed using the Chi-square test and the z-test for proportions. The risk of not achieving the recommended levels of physical activity was analyzed by means of univariate unconditional logistic regressions. For this purpose, the cumulative scores of the items related to the practice of physical activity were considered, these items in turn established a lower figure than those of the WHO recommendations (score ≤ 13 points). From this, the observed sociodemographic factors were incorporated in the regression: gender, age group and socioeconomic level of the participant. Subsequently, a multifactorial logistic regression analysis was performed with the forward procedure, considering a probability of 0.05 as the inclusion criterion and 0.1 as the elimination criterion. The proposed model allowed prediction of the joint effect of exposure to these factors, the size of which was determined by calculating Odds Ratios (OR). Additionally, the goodness of fit of the regression model was considered. All analyzes were performed using SPSS^®^ 21.0 software, considering a significance level of *p* < 0.05.

### Ethical considerations

The participation of each adolescent in the study was voluntary and their data were duly safeguarded. Consents were provided accordingly for each participant and their guardian. These documents were collected by the researchers prior to the application of the self-reports.

## Results

### Participants’ characteristics

Table 1 shows the absolute and relative frequencies of the group examined for each sociodemographic variables considered in the study.

**TABLE 1:** Participants’ characteristics (*N* = 7,263).

		Total 7,263	Male 3, 275 (45.1)	Female 3,988 (54.9)
		n (%)	n (%)	n (%)
Age groups	10–13 years old	3,256 (44.8)	994 (30.4)	1,176 (29.5)
	14–16 years old	3,171 (43.7)	1,439 (43.9)	1,775 (44.5)
	17–20 years old	836 (11.5)	842 (25.7)	1,037 (26.0)
			χ*^2^* = 0.639; *p* = 0.726	
SVI	High	3,479 (47.9)	1523 (48.8)	1812 (47.9)
	Medium	2,099 (28.9)	892 (28.6)	1,116 (29.1)
	Low	1,685 (23.2)	707 (22.6)	910 (23.7)
			χ*^2^* = 1.879; *p* = 0.391	

SVI, school vulnerability index of the educational establishment to which the adolescent belongs.

### Physical activity habits and sociodemographic factors

Table 2 shows that, in general, a small proportion of the total number of adolescents observed engage in some type of physical activity daily. The frequency of young people who engage in some type of physical activity on a daily basis range from 10.3% in the case of practicing physical activity and/or sports with the family, 26.7% who practice some type of physical activity or sport daily in addition to physical education classes. Daily relevant proportions are reached only in the case of the habit related to walking at least 15 min (58.9% of the total).

**TABLE 2:** Physical activity habits based on the sociodemographic factors studied.

		Gender			Age group			SVI		
		Total *n* (%)	Male *n* (%)	Female *n* (%)	10–13 years old *n* (%)	14–16 years old *n* (%)	≥ 17 years old *n* (%)	Low *n* (%)	Medium *n* (%)	High *n* (%)
I do PA or sports with my family.	Never or < once a month	3, 203 (44.1)	1,281 (39.1)^a^	1,946 (48.8)^b^	1,159 (35.6)^a^	1,598 (50.4)^b^	470 (56.2)^c^	664 (39.4)^a^	961 (45.8)^b^	1,576 (45.3)^b^
	1–3 times a month	1,431 (19.7)	609 (18.6) ^a^	830 (20.8)^b^	658 (20.2)^a^	641 (20.2)^a^	127 (15.2)^b^	347 (20.6)^a^	418 (19.9)^a^	675 (19.4)
	1–2 times a week	1,198 (16.5)	563 (17.2)^a^	634 (15.9)^a^	638 (19.6)^a^	438 (13.8)^b^	122 (14.6)^b^	318 (18.9)^a^	315 (15.0)^b^	571 (16.4)^b^
	3–6 times a week	683 (9.4)	373 (11.4)^a^	299 (7.5)^b^	361 (11.1)^a^	260 (8.2)^b^	72 (8.6)^b^	199 (11.8)^a^	197 (9.4)^b^	282 (8.1)^b^
	Daily	748 (10.3)	449 (13.7)^a^	279 (7.0)^b^	440 (13.5)^a^	235 (7.4)^b^	45 (5.4)^c^	157 (9.3)^a^	208 (9.9)^a^	376 (10.8)
			χ*^2^* = 203.234; *p* < 0.001		χ*^2^* = 247.362; *p* < 0.001			χ*^2^* = 50.760; *p* < 0.001		
I play in the park, garden or yard with other children.	Never or < once a month	3,065 (42.2)	1,267 (38.7)^a^	1,819 (45.6)^b^	1,022 (31.4)^a^	1,595 (50.3)^b^	475 (56.8)^c^	725 (43.0)^a^	909 (43.3)^a^	1,437 (41.3)
	1–3 times a month	1,249 (17.2)	534 (16.3)^a^	726 (18.2)^b^	514 (15.8)^a^	618 (19.5)^b^	140 (16.8)^a,b^	275 (16.3)^a^	357 (17.0)^a^	612 (17.6)
	1–2 times a week	1,046 (14.4)	491 (15.0)^a^	546 (13.7)^a^	501 (15.4)^a^	425 (13.4)^b^	115 (13.7)^a,b^	256 (15.2)^a^	294 (14.0)^a^	494 (14.2)
	3–6 times a week	734 (10.1)	396 (12.1)^a^	327 (8.2)^b^	417 (12.8)^a^	260 (8.2)^b^	50 (6.0)^c^	185 (11.0)^a^	191 (9.1)^b^	358 (10.3)^a,b^
	Daily	1,169 (16.1)	586 (17.9)^a^	570 (14.3)^b^	801 (24.6)^a^	273 (8.6)^b^	56 (6.7)^b^	244 (14.5)^a^	348 (16.6)^a,b^	578 (16.6)^b^
			χ*^2^* = 91.079; *p* < 0.001		χ*^2^* = 558.203; *p* < 0.001			χ*^2^* = 13.316*; p* = 0.101		
At recess time I do some sport or PA.	Never or < once a month	3,109 (42.8)	1,041 (31.8)^a^	2,118 (53.1)^b^	925 (28.4)^a^	1,747 (55.1)^b^	533 (63.7)^c^	701 (41.6)^a^	924 (44.0)^a^	1,506 (43.3)
	1–3 times a month	1,097 (15.1)	481 (14.7)^a^	618 (15.5)^a^	472 (14.5)^a^	520 (16.4)^b^	117 (14.0)^a,b^	254 (15.1)^a^	332 (15.8)^a^	511 (14.7)
	1–2 times a week	879 (12.1)	436 (13.3)^a^	443 (11.1)^b^	462 (14.2)^a^	320 (10.1)^b^	75 (9.0)^b^	233 (13.8)^a^	246 (11.7)^b^	400 (11.5)^b^
	3–6 times a week	668 (9.2)	390 (11.9)^a^	263 (6.6)^b^	391 (12.0)^a^	212 (6.7)^b^	38 (4.5)^c^	145 (8.6)^a^	193 (9.2)^a^	320 (9.2)
	Daily	1,511 (20.8)	927 (28.3)^a^	546 (13.7)^b^	1,006 (30.9)^a^	371 (11.7)^b^	74 (8.8)^c^	352 (20.9)^a,b^	405 (19.3)^b^	741 (21.3)
			χ*^2^* = 584.046; *p* < 0.001		χ*^2^* = 807.281; *p* < 0.001			χ*^2^* = 13.629; *p* = 0.092		
I practice some type of PA or sports in addition to the one I do in Physical Education.	Never or < once a month	1,852 (25.5)	603 (18.4)^a^	1,284 (32.2)^b^	658 (20.2)^a^	983 (31.0)^b^	285 (34.1)^b^	359 (21.3)^a^	565 (26.9)^b^	929 (26.7)^b^
	1–3 times a month	1,017 (14.0)	393 (12.0)^a^	634 (15.9)^b^	394 (12.1)^a^	498 (15.7)^b^	113 (13.5)^a,b^	204 (12.1)^a^	292 (13.9)^a,b^	529 (15.2)^b^
	1–2 times a week	1,300 (17.9)	596 (18.2)^a^	702 (17.6)^a^	589 (18.1)^a,b^	545 (17.2)^b^	173 (20.7)^a^	335 (19.9)^a^	359 (17.1)^b^	605 (17.4)^b^
	3–6 times a week	1,155 (15.9)	599 (18.3)^a^	542 (13.6)^b^	537 (16.5)^a^	485 (15.3)^a^	123 (14.7)^a^	330 (19.6)^a^	300 (14.3)^b^	522 (15.0)^b^
	Daily	1,939 (26.7)	1,048 (33.1)^a^	826 (20.7)^b^	1,078 (33.1)^a^	660 (20.8)^b^	142 (17.0)^c^	457 (27.1)^a^	584 (27.8)^a^	894 (25.7)
			χ*^2^* = 371.835; *p* < 0.001		χ*^2^* = 234.038; *p* < 0.001			χ*^2^*^=^ 61.011; *p* < 0.001		
I walk at least 15 minutes per day	Never or < once a month	683 (9.4)	269 (8.2)^a^	419 (10.5)^b^	335 (10.3)^a^	251 (7.9)^b^	83 (9.9)^a.b^	116 (6.9)^a^	206 (9.8)^b^	358 (10.3)^b^
	1–3 times a month	675 (9.3)	265 (8.1)^a^	419 (10.5)^b^	332 (10.2)^a^	282 (8.9)^a,b^	60 (7.2)^b^	140 (8.3)^a^	216 (10.3)^b^	324 (9.3)^a,b^
	1–2 times a week	741 (10.2)	295 (9.0)^a^	451 (11.3)^b^	361 (11.1)^a^	298 (9.4)^b^	77 (9.2)^a,b^	175 (10.4)^a^	214 (10.2)^a^	355 (10.2)
	3–6 times a week	886 (12.2)	413 (12.6)^a^	475 (11.9)^a^	436 (13.4)^a^	349 (11.0)^b^	105 (12.5)^a,b^	229 (13.6)^a^	248 (11.8)^a,b^	411 (11.8)^b^
	Daily	4,278 (58.9)	2,034 (62.1)^a^	2,225 (55.8)^b^	1,791 (55.0)^a^	1,991 (62.8)^b^	512 (61.2)^b^	1,024 (60.8)^a^	1,215 (57.9)^b^	2,032 (58.4)^a,b^
			χ*^2^* = 56.302; *p* < 0.001		χ*^2^* = 41.720; *p* < 0.001			χ*^2^* = 29.450; *p* < 0.001		
Physical activity (PA)	Follows the WHO recommendations	1,155 (15.9)	727 (22.2)^a^	395 (9.9)^b^	736 (22.6)^a^	311 (9.8)^b^	61 (7.3)^c^	263 (15.6)^a^	311 (14.8)^a^	560 (16.1)^a^
	Dont follows the WHO recommendations	6,108 (84.1)	2,548 (77.8)^a^	3,593 (90.1)^b^	2,520 (77.4)^a^	2,860 (90.2)^b^	775 (92.7)^c^	1,422 (84.4)^a^	1,788 (85.2)^a^	2,919 (83.9)^a^
			χ*^2^* 112.521; *p* < 0.001		χ*^2^* 210.796; *p* < 0.001			χ*^2^* 1.782; *p* = 0.410		

PA, Physical activity correspond to sum of the scores obtained in each one of the items of the physical activity scale. ^a,b,c^, z-test for proportions. p < 0.05.

The table also shows the evident differences with respect to physical activity habits considering the sociodemographic factors examined. In each of the contexts, the habitual practice of physical activity was significantly lower among females. This trend is verified in the global score of physical activity habits, where the proportion of women with physical activity habits, is significantly lower than the proportion of males. From the analysis by age group, it can be seen that in general it is the older youth (late adolescence) are less physically active. The proportion of individuals who never or very occasionally engage in physical activity on a monthly basis in or out of school, with family or outside of physical education classes is higher amongst this older age group. When considering all physical activity habits, the analysis indicates that the proportion of adolescents with healthy physical activity habits is lower in the groups defined as middle (14–16 years old) and late adolescence (17–20 years old).

Finally, when examining the habits of young people based on their level of social vulnerability, the results are more disseminated. Differences are observed in the practice of PA or sports in the family: in conditions of increased vulnerability, fewer young people tend to practice some type of PA on a daily basis and those who practically. Something similar occurs with the practice of PA outside the physical education class; those with a medium or high vulnerability condition tend to practice less frequently in the family home. In this case, when physical activity habits are gathered, it is not possible to appreciate differences between the groups.

### Sociodemographic factors associated with physical activity habits

Table 3 shows the multivariate logistic regression to analyze the predictive effect of sociodemographic factors on physical activity habits (R-Square Nagelkerke = 0.123; goodness of fit of model). Physical activity habits below the recommendations were considered as having less than 18 points as the sum of the itemized score, this is associated with fundamental and comparable physical activity habits in the groups. The said score should consider the daily performance of at least three of the five physical activity habits described. According to the results, it is established that gender, age group and vulnerability level are risk factors related to physical activity habits. Accordingly, the risk of physical activity habits below the recommendations is 2.8 times higher in females than in males, 2.4 times higher in the older age groups (14–16 and 17–21 years old) than in the 10–13 age group and 1.1 times more likely in the medium and high vulnerability groups than in the low socioeconomic vulnerability group.

**TABLE 3:** Factors associated with physical activity habits bellow World Health Organization (WHO) recommendations.

	PAH (≤ 13 points score)			
	B	OR	Sig.	CI 95%
Gender (female)	1.019	2.772	<0.001	2.410–3.187
Age group (14–16/ ≥ 17 years old)	0.882	2.415	<0.001	2.137–2.719
SVI (medium/high)	0.103	1.109	0.017	1.018–1.207

SVI, School Vulnerability Index; CI, 95% confidence interval; B, beta coefficient; OR, odds ratio; B, beta coefficient. Note: The reference categories were: gender (male = 0), age group (early adolescence; 10–13 years old = 0), School Vulnerability Index (Low = 0).

## Discussion

This study analyzes physical activity habits and their relationship with sociodemographic factors in Chilean adolescents.

First, the results show in general that a very high proportion of adolescents do not engage in physical activity on a regular basis with the notable exception of walking at least 15 min daily, the proportion of adolescents who were physically active on a daily basis ranged from 10.3 to 26.7%. These findings are certainly consistent with international and national results, which show that a low percentage of young people reach the daily PA recommendations (Giakoni et al., 2021; Van Sluijs et al., 2021).

Second, a relationship is observed between physical activity habits, gender, age and socioeconomic status of adolescents. In all contexts, in and out of school, with parents or with friends, and in the various manifestations represented through play, sports practice, walking and other activities, females’ physical activity habits were, in terms of proportions, significantly less than males. The results also reveal that being a female significantly increases the risk of presenting physical activity habits below international recommendations. These results are consistent with other research that associate women with a lower level of physical activity than men (Bennàsser and Vidal-Conti, 2021), the trend is maintained even when physical activity habits are observed inside the school, in controlled contexts that promote the practice of physical activity at recess (Rodríguez-Rodríguez et al., 2021). The longitudinal study by Abdulaziz Farooq et al. (2021) establishes gender together with socioeconomic level as non-modifiable factors and, in particular, it associates males with more favorable trajectories of healthy physical activity habits. In this regard, Fernández et al. (2017) points out that being a woman at this stage of life is directly related to perceiving there are greater barriers to participating in physical activity. These barriers may even be related to the fact that the supply of physical activity in their environments is not related to their particular needs and preferences; it is suggested that the lack of interest in this regard may be linked to a masculinized model of sport (Pfister and Sisjord, 2013). The systematic review by Camacho-Miñano et al. (2011) indicates that, for women, contexts that combine a favorable climate for the practice of physical activity, characterized by a varied offer of activities of a non-competitive nature, are more suitable. A gendered approach to physical activity promotion policies can therefore contribute to greater development of physical activity among women.

Older adolescents, represented in the middle and late adolescence groups, also showed less adherence to the development of physical activities in the different contexts. The passage from one stage to another constituted a risk factor for the development of Physical activity habits below recommendations. Research agrees that the transition from childhood to adolescence is where a relevant decrease in the frequency and duration of physical activities can be generally appreciated (Patton et al., 2016; Best et al., 2017). The reviews by Corder et al. (2019) and Hayes et al. (2019) examined longitudinal studies carried out from adolescence to adulthood and report that it is during adolescence, particularly in late adolescence, where the most relevant decreases are reported, both in frequency and duration of the physical activity. At the local level, the National Survey of Physical Activity and Sports Habits developed by the Chilean Ministry of Sports (2019), examined children and adolescents aged 5 to 17 years. Among its results, it highlights that the percentage of active subjects, defined as those who perform physical activity at least 60 min a day, decreased significantly with age. The percentage of active individuals goes from 18.1% in children aged 5 to 9 years, to 10.8% in the case of adolescents aged 13 to 17 years. Although this data is more recent, the survey only describes the frequency with which the subjects perform physical activity for at least 60 min daily. Although this information is relevant, it is not possible to establish physical activity habits in terms of everyday activities such as commuting to and from school, family participation, play, use of leisure time and sports practice (Ministry of Sports, 2019). Modifications in physical activity habits may be in part influenced by the use and form of leisure time. In older adolescents, the habitual practice of sports takes on a more competitive nature that increases pressure and subsequent abandonment. Likewise, at this stage, the use of leisure time in sedentary activities associated with socializing with peers, doing homework and the use of screens for video games and surfing the Internet is more frequent (Ferreira et al., 2016; Mikaelsson et al., 2020).

On the other hand, it could be observed that, in less favorable socioeconomic conditions, an important part of the examined physical activity habits was practiced less frequently in adolescents. A lower socioeconomic level, represented in this case by a higher school vulnerability index, constitutes a risk factor for the development of physical activity habits, below WHO recommendations, in Chilean adolescents. It is important to recognize that the school vulnerability index reflects a series of economic, family, housing and environmental conditions that together are associated with a higher level of social vulnerability. The study by Drenowatz et al. (2010) shows that adolescents with lower socioeconomic status develop a less physically activity lifestyle and spend a greater amount of time in sedentary activities. It is important to note that the amount of physical activity in the young people in this study was also associated with nutritional status. This generates a vicious circle since children and adolescents of lower socioeconomic status have a higher prevalence of overweight or obesity, which in turn is related to lower levels of physical activity (Rittenhouse et al., 2011). School vulnerability was ultimately associated with lower possibilities for physical activity. Studies such as Best et al. (2017) establish that, within the predictors of physical activity in adolescents, factors such as having appropriate clothing for sport, or the economic cost associated of access/membership to sports clubs are included. For Chilean children and adolescents, it has been observed that the gap in terms of physical activity habits is largely explained by the existing socioeconomic inequality. This is mainly associated with the opportunities of access and quality of sports infrastructure, the promotion of physical activity within schools and the allocation of resources for its development (Aguilar-Farias et al., 2020).

From another perspective, physical activity habits are related to socioeconomic level based on the physical and social environment in which the adolescent lives. A favorable physical environment considers aspects such as: housing characteristics, urban design, public space, green spaces, etc. From there, young people who receive a favorable physical environment will develop healthier lifestyles, characterized by a greater number of hours of physical activity practice and an increase in such practice within the family (Bennàsser and Vidal-Conti, 2021). Along the same lines, the research by Rodríguez-Romo et al. (2013) shows that residing in areas considered favorable, characterized by access to recreational facilities, walking possibilities and low residential density, were positively associated with the practice of physical activity. The WHO, in this regard, has established the need for each country to implement and create favorable environments for health that ultimately increase PA in school-age adolescents (World Health Organization [WHO], 2016). In this regard, public policies such as facilitating access to parks or playgrounds, increasing safety, and improving the lighting of outdoor spaces are measures that promote the practice of physical activity in more vulnerable adolescents (Rydenstam et al., 2020). On the other hand, the study by Christian et al. (2016) evaluated the impact of subsidizing access to physical activity and sport facilities and services. Based on a voucher scheme, they sought to promote the practice of physical activity among socially vulnerable adolescents; the results of this study show that the use of these vouchers generated an improvement in the condition and level of physical activity in adolescents. Regarding the social environment, perceived social support from parents and friends is considered a factor that influences the physical activity habits of adolescents (Lisboa et al., 2021), active friends and parents promote greater physical activity practice (Mikaelsson et al., 2020). School peers also play an important role, the influence of peers on physical activity habits in children and adolescents has been demonstrated. Adolescents tend to be active when their friends are active and these in turn, present a healthy nutritional status (Rittenhouse et al., 2011).

Finally, the predictive factor analysis established that being female, being older and living in conditions of social vulnerability significantly increase the risk of presenting physical activity habits below international recommendations. These findings coincide with the findings of Jiménez Boraita et al. (2021) who established, in Spanish adolescents, that older individuals, women and having a low/medium socioeconomic level, together with other factors, were predictors of a low level of physical activity. Ricardo et al. (2022) analyzed physical activity habits data from 64 countries in the southern hemisphere. Their results showed a prevalence of PA 6.7 percentage points higher in boys. Likewise, the boys presented a ratio associated with the prevalence of PA 1.58 higher than the girls. The gap between boys and girls remained even in countries with high human development indices and low gender inequality indices. Likewise, the study by Viciana et al. (2016) recognizes within the personal factors that gender and age significantly influence the practice of moderate and high intensity physical activity. The factors examined are undoubtedly related to the practice of physical activity, as well as to other health risk factors such as obesity. Recognizing the relationship of sociodemographic factors and physical activity habits is fundamental in countries like Chile, a country with significant levels of inequality (Mieres Brevis, 2020), since it will allow the creation of guidelines adapted to a specific local context (Hämäläinen et al., 2020) for Chilean adolescents.

### Strength and limitations

Among the strengths of this study are the use of a validated instrument and the use of standardized measurement criteria. Additionally, the representativeness and socioeconomic diversity of the sample in terms of its size, as well as its selection method, all stand out to as major positives. Finally, we can highlight that the predictive effect of sociodemographic factors was analyzed based on a varied set of physical activity habits and not only on the basis of the frequency and duration of physical activity as in the majority of other studies scrutinized.

There are some limitations in this study. First, that the research results are based on self-report of adolescents, this may be related to the participants’ responses being biased by social desirability condition. Secondly, it is assumed that the socioeconomic level described is established on the basis of the school vulnerability index, which, although it is a measure that summarizes the socioeconomic condition of schoolchildren, may not necessarily represent the reality of each adolescent examined.

## Conclusion

Our study shows generally physical activity habits in Chilean adolescents, were below international recommendations. Despite the differences that could be appreciated from the sociodemographic factors studied, the group examined is characterized by a very low proportion of young people who practice daily physical activity in and out of school, with friends or family. Walking at least 15 min was the physical activity habit with the highest adherence. Age and gender were the factors with the greatest predictive effect; however, all the factors examined together were able to predict the physical activity habits of adolescents and ultimately provided insight into the factors that trigger conditions of inequality with respect to the development of healthy lifestyle behaviors. These findings together with others in a similar vein can contribute greatly to the design of programs or public policies that promote an active lifestyle in adolescents.

## Data availability statement

The raw data supporting the conclusions of this article will be made available by the authors, without undue reservation.

## Ethics statement

The studies involving human participants were reviewed and approved by Committee of Bioethics of the Pontifical Catholic University of Valparaíso (BIOEPUCV-H 427-2021) issued in April 2017. Written informed consent to participate in this study was provided by the participants’ legal guardian/next of kin.

## Author contributions

SF-U, AR, and CF-R originally designed the study. AR and JCO collected the data. SF-U, HC-Q, and AR made the data preparation. SF-U, JP-C, and CF-R were responsible for statistical analyses and double-checked by AR and CF-R. S-FU and CC-C were in charge of drafting of the manuscript. All authors provided support for data interpretation, feedback on drafts, and approved the final manuscript.
